# A strategy for enhanced circular DNA construction efficiency based on DNA cyclization after microbial transformation

**DOI:** 10.1186/s12934-015-0204-x

**Published:** 2015-02-12

**Authors:** Ying-Ying Guo, Zhen-Yu Shi, Xiao-Zhi Fu, Jin-Chun Chen, Qiong Wu, Guo-Qiang Chen

**Affiliations:** MOE Key Lab of Bioinformatics, Department of Biological Science and Biotechnology, School of Life Science, Tsinghua-Peking Center for Life Sciences, Tsinghua University, Beijing, 100084 China; Center for Nano and Micro Mechanics, Tsinghua University, Beijing, 100084 China; Synthenome.com, Dingley Village, VIC3172 Australia

**Keywords:** Synthetic biology, DNA assembling, Circular DNA, DNA ligation, Sequence and ligation independent cloning

## Abstract

**Background:**

With the rapid development of synthetic biology, the demand for assembling multiple DNA (genes) fragments into a large circular DNA structure in one step has dramatically increased. However, for constructions of most circular DNA, there are two contradictions in the ligation/assembly and transformation steps. The ligation/assembly consists of two different reactions: 1) the ligation/assembly between any two pieces of a linear form DNA; 2) the cyclization (or self-ligation) of a single linear form DNA. The first contradiction is that the bimolecular ligation/assembly requires a higher DNA concentration while the cyclization favors a lower one; the second contradiction is that a successful transformation of a ligation/assembly product requires a relatively high DNA concentration again. This study is the first attempt to use linear plasmid and Cyclization After Transformation (CAT) strategy to neutralize those contradictions systematically.

**Results:**

The linear assembly combined with CAT method was demonstrated to increase the overall construction efficiency by 3–4 times for both the traditional ligation and for the new *in vitro* recombination-based assembly methods including recombinant DNA, Golden Gate, SLIC (Sequence and Ligation Independent Cloning) and Gibson Isothermal Assembly. Finally, the linear assembly combined with CAT method was successfully applied to assemble a pathway of 7 gene fragments responsible for synthesizing precorrin 3A which is an important intermediate in VB12 production.

**Conclusion:**

The linear assembly combined with CAT strategy method can be regarded as a general strategy to enhance the efficiency of most existing circular DNA construction technologies and could be used in construction of a metabolic pathway consisting of multiple genes.

**Electronic supplementary material:**

The online version of this article (doi:10.1186/s12934-015-0204-x) contains supplementary material, which is available to authorized users.

## Background

The rapid developing synthetic biology has an increasing need for assembling multiple genes or operons into a large DNA fragment consisting of multiple metabolic pathways [1-4]. At present, three major DNA assembly techniques were commonly used: The first one is the traditional recombinant DNA technology based on the type II restriction endonucleases, BioBrick and BglBrick are two representatives, mutiple DNA fragments with standardized flanking sequences allow assembly to achieve via a simple and standardized restriction/ligation procedure [5,6]. However, the BioBrick and BglBrick techniques cannot be used for seamless assembly due to the use of restriction enzyme(s). The assembly becomes laborious when applied to a pathway involving mutiple genes. The challenge of multiple DNA fragment assembly has led to the emerge of Golden Gate method, a parallel one-pot, one-step 5 min technique to seamless assemble larger constructs [7,8]. Golden Gate method had been successfully used to assemble a 32 kb gene cluster [8].

The second DNA assembly technique is *in vitro* recombination method based on single-strand overhangs generated by single-strand exonucleases or incomplete PCR (Polymer Chain Reaction), typically represented by OE-PCR (Overlap Extension-PCR). The method is commonly used *in vitro* to assemble 0.5 to 5 kb DNA fragment [9]. For multiple DNA assembly by the second method, three more improvements including In-Fusion, SLIC and Gibson have been made [10-12]. All three improvements use a proprietary enzyme mixture to assemble any fragment with 15+ bp sequence overlap. The last common method is USER (uracil-specific excision reagent) cloning, which first introduces at least one uracil in the PCR process, followed by excision of uracil using uracil DNA glycosylase [13]. The resultant abasic site(s) cleaved by an AP-lyase, leaving 30 overhangs annealed with the complementary overhang of a fragment sharing the same overlap sequence [13].

The third major DNA assembly technique is the *in vivo* recombination that requires certain recombination systems operating inside the cells [14,15], represented by the yeast homologous recombination developed by J. Craig Venter Institute [16,17]. It can be used to assemble the entire circular synthetic *Mycoplasma. genitalium* genome in one step from 25 DNA pieces approximately 24 kb in size [18]. For genome-sized assembly, yeast is not the only prefer host; recently the complete assembly of chromosomes was also achieved in *Bacillus* by Itaya et al. [19]. The 3500 kb of *Synechocystis* DNA was inserted into *B. subtilis* from some 100 kb + long starting DNA fragments [20]. Except for the extreme case of blunt ligation, both the first two assembly methods rely on the annealing of single strand DNA overhangs. Some of these technologies were reported to be successful in assembling 4–6 fragments routinely such as the Gibson isothermal assembly [12]. Nevertheless, they suffer from demanding operations and constrains on fragment lengths mentioned in their protocols. Therefore, multiple fragment assembly is still challenging and requires improvement on efficiency.

**Figure 1:**
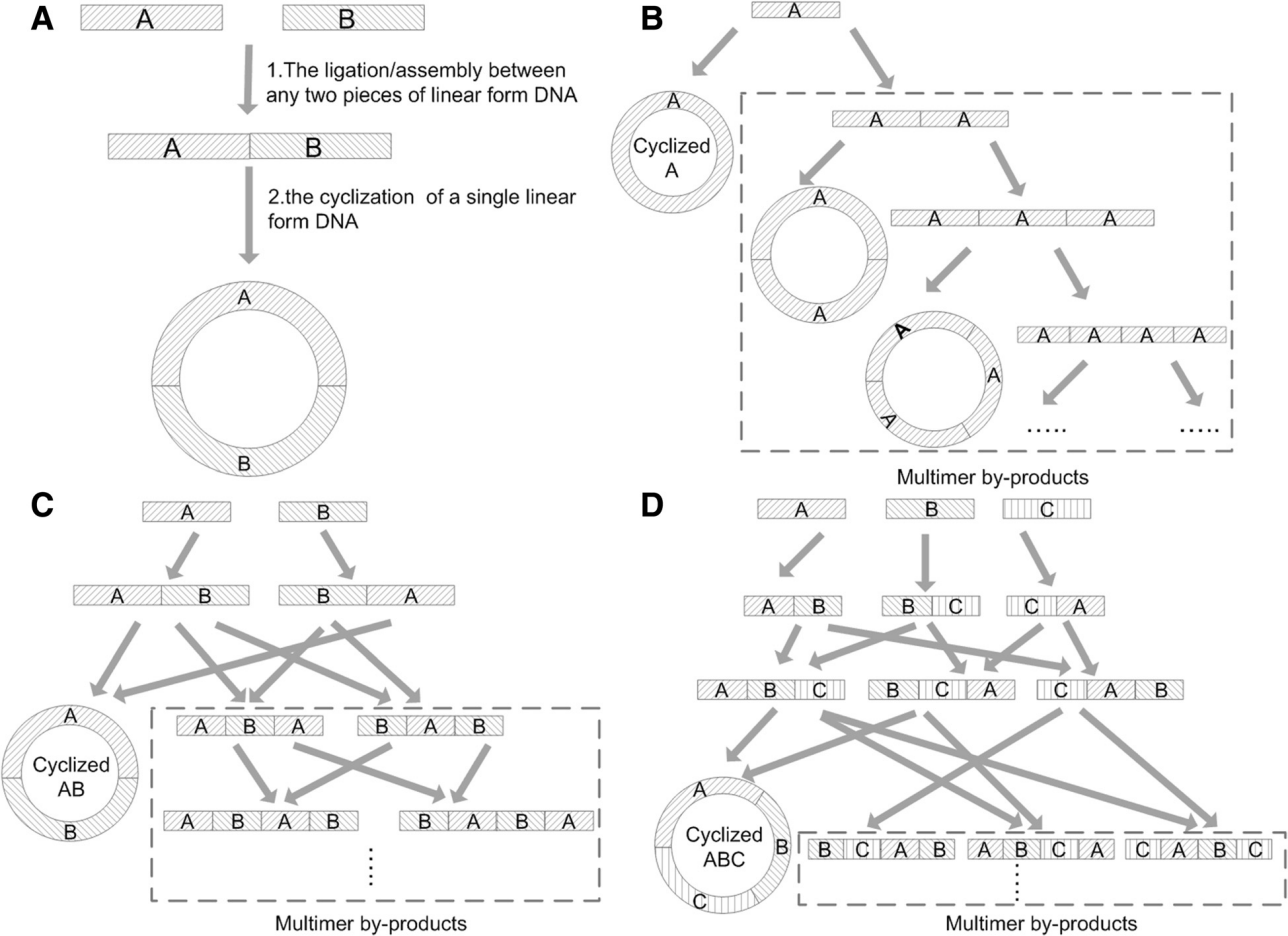
**Mechanism of the contradiction between bimolecular ligation and cyclization and its resulting by-production formation mechanisms. A)** In any construction of circular DNA from linear forms, there are both bimolecular ligation/assembly and monomolecular cyclization. **B)** Multimer by-product formation mechanism in single molecule cyclization. **C)** By-product formation mechanism when circular plasmids are constructed from two fragments. **D)** This contradiction becomes more obvious when more fragments are involved in ligations.

Apart from strategies to improve ligation and *in vitro* recombination efficiency, two contradictions in most circular DNA construction procedures regarding the DNA concentration have been mostly neglected so far [7,17]. The first contradiction is that the bimolecular ligation/assembly and the monomolecular cyclization require different DNA concentrations. In any construction of circular DNA from linear forms, there are both bimolecular ligation/assembly and monomolecular cyclization (Figure 1A). Specifically, the bimolecular ligation/assembly between two linear DNA fragments needs a high reagent concentration, yet the monomolecular cyclization of linear DNA into circular form requires low concentration to reduce formations of multimer by-products. The second contradiction is that DNA transformation including both electroporation and chemical approach usually requires a relatively high DNA concentration, yet the cyclization step requires lower concentration as mentioned previously [12]. Considering all the three steps, most ligation/assembly of linear DNA fragments into circular forms is recommended to perform at a compromised DNA concentration. Therefore, the overall construction yield is sacrificed due to the formation of multimer by-products at the cyclization step [7]. In addition, more fractions of multimers will be formed when more linear fragments are assembled/ligated in one reaction (Figure 1B-D). This issue is even more important for multiple fragments DNA ligation/assembly such as Golden Gate assembly and Gibson isothermal assembly. However, few studies were devoted to solve the concentration dilemma caused by the above mentioned three steps so far.

The current study focuses on finding a solution to the dilemma: if the cyclization step is not in between the other two steps that require a high DNA concentration, the dilemma should be avoided. This solution will have three advantages: there is no by-product if all DNA fragments are mixed at an equal molar ratio when the target ligation/assembly product is linear form DNA with each junction designed to be specific (Figure 1C); secondly, the absence of by-product further allows even higher concentration for ligation/assembly and subsequently for an efficient transformation; thirdly, if an efficient *in vivo* cyclization after transformation can occur, the transformation will dilute and even separate the DNA physically by cell walls to allow higher yield of *in vivo* cyclization of linear DNA inserted into the cells with less oligomer by-products than cyclizations performed at a higher ligation concentration.

Alternatively, there is also a way to avoid cyclization if linear plasmids are used. The linear lambdoid phage N15 is one of the best studied *E. coli* phages due to its unique linear form, whose linear ends are protected by telomerase attached hairpins [21]. A series minimized N15 phages were developed and reported to improve the cloning efficiency of large DNA fragments [22]. They are candidates for constructing plasmids without cyclization.

A linear ligation/assembly with Cyclization after Transformation (CAT) strategy mentioned above could provide a way out of the dilemma, it could be especially useful for multiple fragments DNA ligation/assembly.

## Results

### Unidirectional linear ligation system and grouped assembly

Since the SLIC and Gibson assembly methods are specific at every single DNA junction unless repetitive DNA is assembled. The first goal of this study was to establish a proper ligation strategy for traditional type II restriction endonucleases based methods.

**Figure 2:**
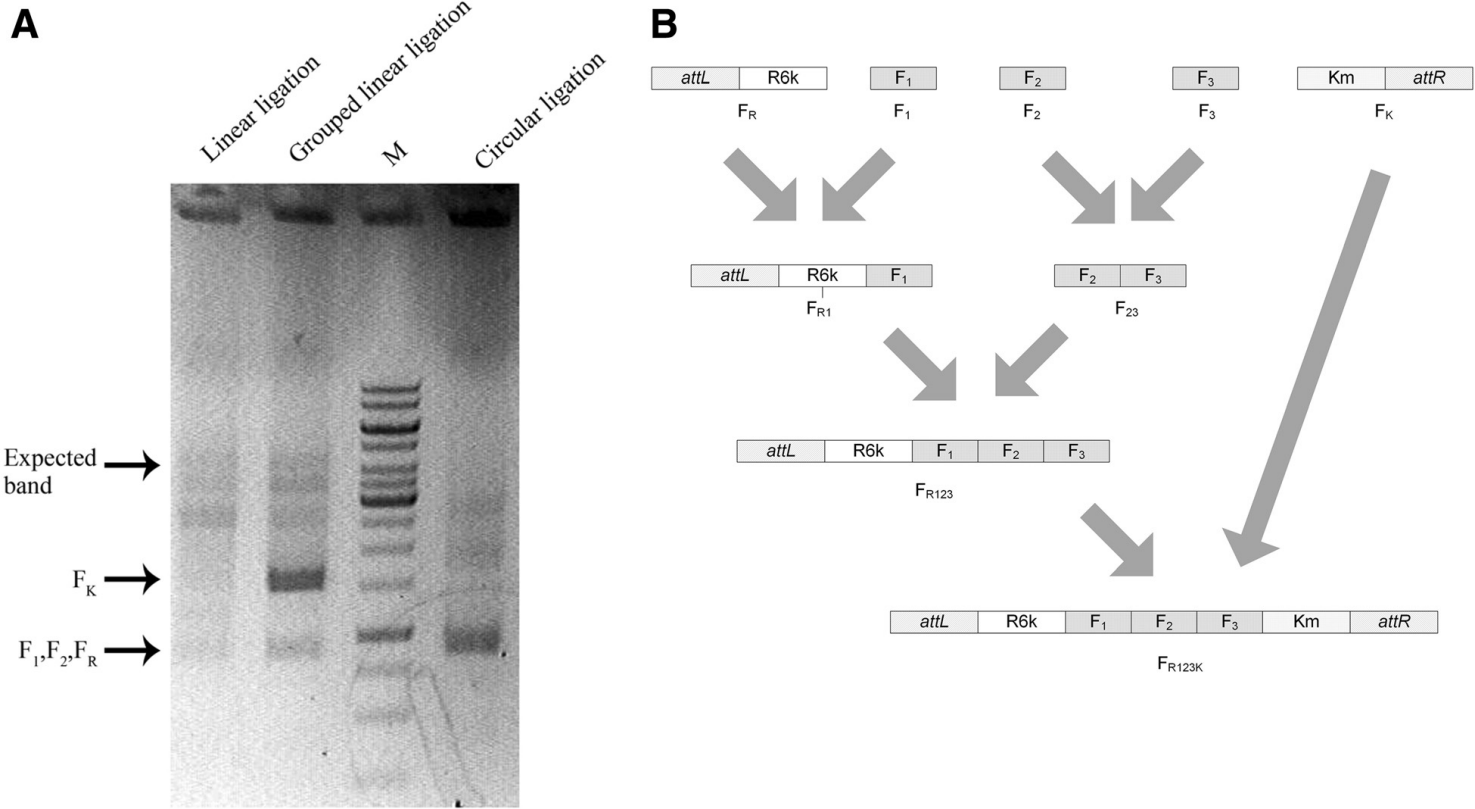
**Description of grouped linear ligation and comparison of ligation efficency of circular ligation, linear ligation and grouped ligation. A)** Gel Electrophoresis of linear ligation, grouped linear ligation and circular ligation products for four fragments. **B)** Description of grouped linear ligation of F_R_, F_1_, F_2_, F_3_ and F_K_.

An *in vitro* linear ligation system based on the type II restriction endonucleases was developed to provide specificity to the theoretically by-product free ligation. In this *in vitro* linear ligation system, five restriction endonucleases produced non-palindromic 3’ sticky ends with the customized triplet NNN [23-26], 33 DNA fragments with 64 properly designed overhangs could theoretically be ligated into one fragment in one mixture. A 4 fragments ligation experiment containing F_1_ (935 bp), F_2_ (948 bp), F_R_ (921 bp) and F_k_ (1598 bp) was designed, the 4 fragments were mixed together at the same molar concentration with the T4 ligation system. After the 2 hours ligation, the mixture was treated with SDS at 75°C for 20 min before electrophoresis to remove ligase from ligated DNA. The electrophoresis showed that more expected ligation products were obtained using the linear ligation system than that of the circular ligation system when the same DNA molar concentration was added for every ligated fragment used in the two systems (Figure 2A). As expected, the sequencing results of some incorrectly assembling plasmids by the circular ligation demonstrated the formation of multimer by-products (data not shown). Of course these incorrectly assembled products were expectable.

Although the designed sticky ends created by 5 endonucleases could theoretically be ligated in only one way, unexpected annealing of non-complementary sticky ends were always observed when more than two fragments were mixed together. In a previous study, T to G and A to C mismatches were reported to occur in unidirectional ligation [7]. In our study, similar phenomenon was observed when the incorrectly assembled plasmids were sequenced.

Therefore, a computer program termed StickyEndDesigner was developed to reduce mismatching phenomena during the primer design process. A copy of this software can be freely downloaded from http://stickyenddesigner.codeplex.com/. This software was designed to avoid T or A as the last base in the sticky ends, because the T or A base easily leads to mismatch in the annealing process. With all possibilities considered, the software generates only 8 sets of sticky ends that satisfied the requirements above. Hence, the previous 4 DNA fragment ligation process was modified to use 3 pairs of software-designed triplets. In addition, a grouped ligation procedure was applied with the hope that physical isolation of subgroups may show a better performance in reducing mismatch phenomenon (Figure 2B). Impressively, a denser target band was observed in the electrophoresis for the final round of grouped ligation mixture (Figure 2A, lane 2), indicating a reduced unexpected ligation, this was accompanied by increasing number of correctly constructed plasmids.

Different ligases and buffers were compared during the 4 fragments ligation experiments containing F_1_ (935 bp), F_2_ (948 bp), F_R_ (921 bp) and F_k_ (1598 bp). The 4 fragments were mixed at the same molar concentration with the different ligation systems preapared above. After the 2 h ligation, the ligation efficiency was studied after the mixture was treated with SDS at 75°C for 20 min before electrophoresis. Solution I containing T4 ligase was revealed to be the most suitable for the ligation. In addition, *in vitro* linear ligation efficiency was also dependent on DNA fragment purity (data not shown). For example, the ligation efficiency was much higher if the DNA fragment was cloned by Phusion polymerase and purified via gel extraction compared with that of its cloned by Pfu polymerase and purified by solution extraction.

### Linear plasmid as an alternative choice to avoid cyclization

**Figure 3:**
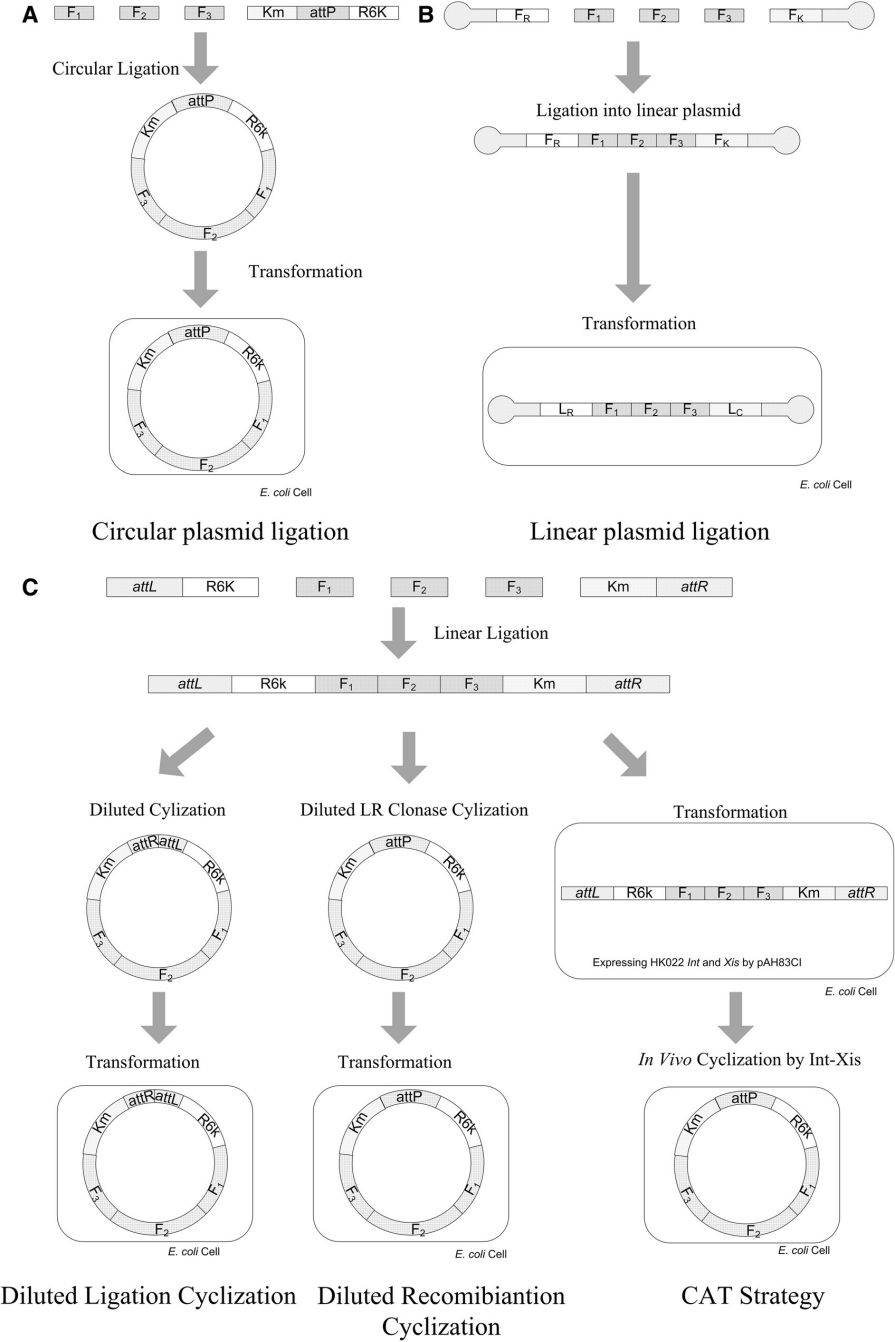
**Schematic description of various attempts to avoid the contradiction on bimolecular ligation/assembly and monomolecular cyclization. A)** Conventional circular ligation, **B)** Ligation into linear plasmid. No cyclization is required to make the plasmid stable and thus the contradiction was avoided. **C)** Linear ligation and diluted self-ligating cyclization (First branch from the left), linear ligation and *in vitro LR* clonase cyclization (Second branch from the left) linear ligation coupled with *in vivo* cyclization (Third branch from the left).

As a shortcut to reproducible *E. coli* plasmid assembled from linear fragments, a linear plasmid is apparently an attractive option to avoid the multimer issue associated with circular plasmids (Figure 3A-B). Therefore, the efficiency of linear plasmids ligation from multiple fragments was investigated by ligating 1, 2 or 3 gene fragments into a linear plasmid (Figure 3B). It was found very difficult even to insert more than one DNA fragment in between the F_R_ and F_K_. Since the F_R_ and F_K_ were the purified digestion products of plasmids with sizes up to 10 Kbp, their molar concentrations are much lower than the PCR products so that the inserting fragments have to be diluted to match their concentration requirement to avoid by-product formation.

### Two traditional *in vitro* cyclization strategies

The *in vitro* linear ligation system was developed in our study to prevent the multimer by-product formation. However, the linear ligation products formed *in vitro* could not replicate in *E. coli* due to their linear nature. Therefore, an efficient cyclization strategy must be established. We further evaluated the *“in vitro* cyclization” by comparing with two traditional strategies.

The two traditional *in vitro* methods are “diluted ligation cyclization” and “diluted recombination cyclization”. In the “diluted ligation cyclization” method, the yield of cyclization was determined based on the cases of 4 and 6 DNA fragments assembling into circular plasmids with controls of corresponding grouped circular ligations (Additional file 1: Table S1). Results showed that “diluted ligation cyclization” method resulted in a lower colony number and a higher accuracy than that of the circular cyclization (Table 1). The second strategy was termed “diluted recombination cyclization”, the highly efficient site-specific recombination also turns linear DNA into circular one (Figure 3C). *LR* clonase II was used to cyclize the grouped ligation product of 6 DNA fragments with 40 colonies obtained on a selective plate, of which, only 3 were eventually found to be the correct ligation form (Table 1).

**Table 1:** **Number of transformants formed using conventional** ***in vitro*** **circular ligation system,** ***in vitro*** **diluted self-ligation cyclization,** ***in vitro*** **self-recombination cyclization, and CAT** ***,*** **respectively**

**Ligation**	**4 DNA fragments**	**6 DNA fragments**
**Conventional** ***in vitro*** **circular ligation system (S17-1λ** ***pir*** **)**	204(16/96)	60(2/60)
***In vitro*** **diluted self-ligation cyclization (S17-1λ** ***pir*** **)**	162(42/96)	22 (12/22)
***In vitro*** **self-recombination cyclization (S17-1λ** ***pir*** **)**	----	40(3/40)
**CAT strategy (S17-1λ** ***pir∆attB*** **HK022 pAH83CI)**	200(80/96)	42(36/42)

### CAT (Cyclization after transformation) strategy

The two traditional *in vitro* cyclization strategies were not as efficient as expected. Therefore, a new CAT (Cyclization after Transformation) strategy was developed. In CAT, the ligation product *E. coli* S17-1 λ*pir*Δ*attB*_HK022_ harboring pAH83CI was transformed with the ligation product (See Bacterial Strains and Mediums) and the ligation product would to be cyclized via the *attL-attR in vivo* recombination system (Figure 3C). Four and six fragments were assembled based on the CAT strategy above, as a result, 80 in 96 and 36 in 42 clones were demonstrated to be properly assembled via PCR screening of every junction, respectively, and followed by the supporting sequencing results (Table 1). Both transformation yield and DNA assembly accuracy based on the CAT strategy outperformed those of the circular ligation and diluted cyclization. It was demonstrated that the efficiencies of grouped unidirectional ligation coupled with CAT strategy are more efficient and precise than that of the conventional circular ligation, “*in vitro* diluted self-ligation cyclization” and “*in vitro* self-recombination cyclization” (Table 1). As expected, less colony numbers containing 6 properly assembled fragments were formed compared with those consisting of 4 fragments assembly. In order to further explore the advantages of this CAT strategy on multiple fragments assembly, nine DNA fragments assembly was performed (Additional file 2: Table S2). The number of successful transformants selected by kanamycin was only 16, and 2 of them were found to be assembled in the correct order via PCR screening and DNA sequencing. Colony PCR was used to study ligation accuracies. 96 randomly selected transformants were investigated if the colony number exceeded 96. After screening by colony PCR, random colonies were selected to do plasmid extraction. The extracted plasmids were analyzed by endonuclease digestion and DNA sequencing. It was found that all the sequencing results were consistent with the colony PCR ones. Also, the incorrect constructed plasmids were sequenced, the sequencing results showed that the incorrect assembly could mainly be contributed to the ligation mismatching.

### CAT-Recombinant DNA: Recombinant DNA using CAT

**Figure 4:**
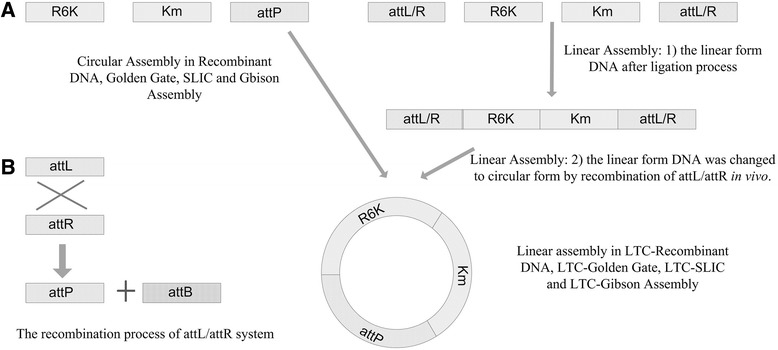
**The experimental comparison between non-CAT and CAT strategy. A)** The non-CAT control group assembled 3 fragments into a circular form *in vitro* and thus engaged 3 *in vitro* ligation/assembly reactions (left). CAT strategy assembled 4 fragments with *attL* and *attR* as the linear ends in *in vitro* ligation/assembly, thus also engaged precisely the same three *in vitro* ligation/assembly reactions (right). **B)** The cyclization mechanism. *attL* and *attR* recombines and produces *attP*, and thus the final products in CAT strategy are the same as the final products in non-CAT control group.

In order to demonstrate that CAT strategy can improve the efficiency when it was combined with other assembly technique(s), CAT was first applied in existing recombinant DNA method. Since CAT strategy always has its linear loose ends enclosed by *in vivo* recombination, the three *in vitro* linear ligation steps of CAT strategy were identical to the 3 ligations of the non-CAT ligation control. That is why CAT strategy with 4 fragments should be compared to non-CAT strategy with 3 fragments (Figure 4A-B). In order to maintain the same plasmid concentration level in both CAT and non-CAT controls, both groups have the same total DNA concentration. At 2 pM total DNA molar concentration, CAT strategy enhanced 3.48 folds efficiency compared with the non-CAT control (Table 2 and Figure 4A).

**Table 2:** **Results of the comparisons between CAT strategy and non-CAT control**

**Ligation**	**0.5 pM**	**1 pM**	**2 pM**
**Circular Recombinant DNA**	38 (35/38)	184 (44/48)	304 (40/48)
**CAT** **- Recombinant DNA**	88 (44/48)	364 (35/48)	804 (42/48)
**CAT** **/non-** **CAT** **× 4/3**	3.07	2.10	3.70
**Circular Gibson Assembly**	84 (37/48)	168 (32/48)	284 (32/48)
**CAT** **- Gibson Assembly**	140 (40/48)	308 (33/48)	624 (38/48)
**CAT** **/non-** **CAT** **× 4/3**	2.40	2.52	3.48
**Circular SLIC**	66 (20/48)	142 (25/48)	276 (28/48)
**CAT** **-SLIC**	124 (38/48)	212 (41/48)	486 (40/48)
**CAT** **/non-** **CAT** **× 4/3**	4.76	3.26	3.48
**Circular Golden Gate**	N/A	4 (3/4)	25 (10/25)
**CAT** **- Golden Gate**	N/A	132 (40/48)	248 (29/48)

The comparisons between CAT and non-CAT control (explained in Figure 4) were presented by the colony number and correct colony number. CAT/non-CAT is the correct colony number in CAT over the correct colony number in non-CAT.

### CAT-Golden Gate

Golden Gate method has a low overall efficiency when the concentration is low. We repeated 3 times yet were not able to obtain reliable data at 0.5 pM total DNA molar concentration. When the total concentrations are 1 pM and 2 pM, respectively, however, CAT strategy showed exceptional 24.44 times and 19.98 times more efficient compared with the non-CAT controls, respectively (Table 2).

### CAT-SLIC

In order to demonstrate that the CAT strategy can truly improve the efficiency for DNA assembly, and it was possible to apply to SLIC method. Similar to the recombinant DNA (ligation) method, the overall efficiency of CAT turned out to be 3.48 times of the non-CAT one at 2 pM total DNA molar concentration (Table 2).

### CAT-Gibson assembly

When CAT strategy was applied to Gibson isothermal assembly, similarly, an overall efficiency as 3.48 times high as the non-CAT control was observed at 2 pM total DNA molar concentration (Table 2).

### Construction of optimized precorrin-3A production pathway from precursor 5-aminolevulinic acid

**Figure 5:**
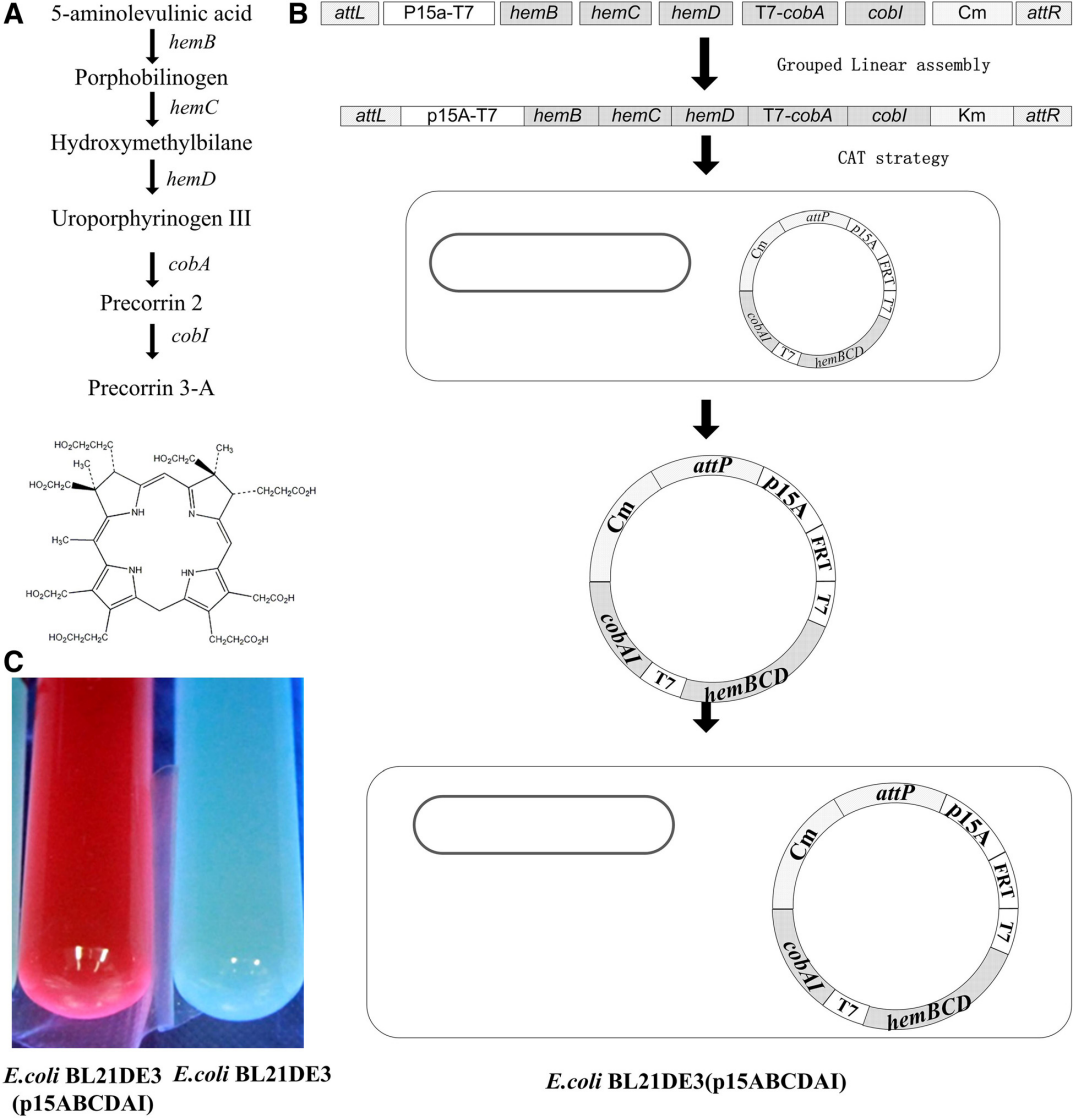
**Construction and verification of precorrin-3A synthesis pathway. A)** Schematic diagram of precorrin-3A pathway. **B)** Construction of *E. coli* strains BL21DE3 (p15ABCDAI). C): The color change of the culture supernatant of *E. coli* strains BL21DE3 (p15ABCDAI). These strains were cultured in LB medium plus with 1 g/L 5-aminolevulinic acid.

Precorrin-3A pathway was constructed with linear ligation coupled with CAT strategy as an application in metabolic engineering. Aerobic bacterium *Psudomonas denitrificans* produces corrins via an oxygen-dependent pathway [27]. Precorrin-3A is the first of the tetrapyrrolic intermediates committed solely to vitamin **B**_12_ biosynthesis. From the precursor 5-aminolevulinic acid to precorrin-3A, there are 5 steps including 5 genes *hemBCD, cobAI* (Figure 5A)*.* The five genes were constructed into the plasmid p15ABCDAI with the linear ligation coupled with CAT strategy, and then *E. coli* strain BL21DE3 was transformed with p15ABCDAI (Figure 5B). The recombinant strain was cultured in LB medium supplemented with 1 g/L 5-aminolevulinic acid as a precursor for production of precorrin-3A. When grown in LB with 1 g/L 5-aminolevulinic acid, the fermentation broth supernatants of recombinant strain developed a red color and exhibited a bright red fluorescence at 301 nm as reported for precorrin-3A (Figure 5C) [28].

## Discussion

### Grouped ligation can avoid DNA assembling mismatch

Circular ligation always comes along with the formation of multimer by-products (Figure 1B-D). On the other hand, the absence of multimer by-products indicated no occurrence of circular ligations. Linear ligation offers a possibility to overcome this contradiction (Figure 3C).

As long as every DNA fragment has an equal molar concentration in the mixture, and the ligation yield of every ligation process is 100%, the theoretical ligation yield should be 100%. To precisely address the effect of each fragment on ligation yield, theoretical models (http://www.synthenome.com/StickyEndDesigner.zip) were developed to study effects of concentrations of the DNA fragments in the ligation mixture on linear ligation yield. It was found that over-dose of the first and the last fragments, namely F_R_ and F_K_ did not affect ligation yield, while other DNA fragments in between must have an equal molar ratio to avoid ligation by-products.

However, ligation yield was always below 100% under experimental conditions due to inadequate ligations and unexpected ligations (Figure 2A). Many studies focused on unexpected ligation resulted from mismatching ligation, such as the mismatches of T to G and A to C [7]. It is useful to properly design sticky ends so that unexpected ligation can be significantly reduced. However, the designed sticky ends did not significantly reduce mismatches (data not shown). Therefore, the method of grouped ligation into a linear fragment with a free end was developed. The method was found the most efficient in reducing the ligation mismatches (Figure 2B). As grouped ligation isolates fragments that are not designed to ligate, and thus lead to reduction on unexpected ligations. Furthermore, parts of the unexpected ligation may be attributed to unexpected digestion of the single-strand overhangs [29]. It could be expected that unwanted digestions can be significantly reduced when highly pure reagents were used. The single strand annealing nature determines that unspecific annealing would be also an issue for *in vitro* the recombination methods such as SLIC and Gibson isothermal assembly. This study also demonstrates that the grouped ligation/assembly can enhance SLIC assembly efficiency.

### CAT strategy takes advantages of DNA concentration conditions in both ligation and transformation

Unidirectional ligation/assembly requires a DNA concentration to be high enough to allow efficient bimolecular ligation/assembly, transformation will benefit from a high DNA concentration of the ligation/assembly mixture. However, the challenge for transforming linear ligation products into a host is the difficulty to cyclize them rapidly and efficiently *in vivo* to form stable and reproducible circular DNA form. In order to obtain a high cyclization efficiency, site-specific recombination was selected as the cyclization strategy, as this *attB*-*attP* recombination catalyzed by Int or Int-Xis has very high efficiency (Figure 3C, right hand) [30]. When *E. coli* DH5 alpha harboring pInt-ts was transformed with plasmid pAH63 [31], the number of resulting successful transformants could match that of *E. coli* S17 λ*pir*. That is to say, if the transformation efficiencies of linear and circular DNA are similar, the *attB*-*attP in vivo* recombination may be close to 100% (Additional file 3: Figure S1) [31]. Therefore, the *in vivo* cyclization does not result in decrease in efficiency. During the transformation, the transformed linear ligation products are naturally diluted by competent cell mixtures and finally isolated physically by cell walls. This provides a situation that favors a much higher yield of self-cyclization. Our results demonstrated that circular ligation of six fragments showed a lower efficiency than that of the linear ligation coupled with *in vivo* cyclization of six fragments (Table 1). Therefore, the grouped ligation coupled with the *in vivo* cyclization process can take advantages of all procedures including: a close to 100% theoretical yield of linear ligation, a dilution during transformation, and a close to 100% yield on *in vivo* lambda attachment sequences recombination, as well as a final high self-cyclization yield (Figure 3C, right hand). Furthermore, this strategy showed enhanced efficiency in ligating at least nine fragments (data not shown) than that of the traditional circular approaches.

### CAT strategy increases overall construction efficiency for existing circular DNA construction methods

In order to demonstrate that the CAT strategy can improve the efficiency for all known existing circular plasmids construction methods, it was applied to 4 most widely used methods, i.e. Recombinant DNA, Golden Gate, SLIC and Gibson isothermal assembly (Table 2). Since it is a challenge to obtain highly concentrated DNA, a comparison was made using CAT strategy and non-CAT control based on same total DNA concentration to ensure that there is no additional difficulty in preparing DNA for CAT strategy. As a result, the multiplier 4/3 is applied to the CAT/non-CAT number to present the true difference in efficiency. It was found that CAT strategy generated a 3.55 ± 0.13 times correct colonies of the original technologies for all technologies except Golden Gate. And this stable 3.55 times may indicate that there is at least 71.8% by-products in the circular ligation/assembly products for the three fragments when each fragment is 0.67 pM in the ligation/assembly mixture. Another assembly technique including recombination of *attL/R* for assembling multiple fragments should also benefit from CAT strategy [32].

### CAT strategy is easy to use together with most existing circular construction technology

Any existing circular construction system can easily adopt CAT strategy by splitting the plasmid backbone into 2 fragments and this will not affect the ligation/assembly of the other fragments/inserts. When *attP* scar is allowed to appear between inserts and plasmid backbone, a 5-time higher efficiency could be possible, which means, the efficiency of the CAT strategy was 5 times as high as the conventional *in vitro* cyclization (Table 1). Although the CAT strategy requires the *in vivo* cyclization step, this step is identical to typical electroporation because the recombination occurs *in vivo* without even the need for heat induction. The only additional *in vitro* operation is the preparation of an additional fragment. Furthermore, as discussed in the section above, no additional efforts in concentrating DNA is required to adopt CAT strategy.

## Conclusion

Synthetic biology often requires the assembly of many fragments at one time. Low concentration and high by-products ratio are the two major challenges for such assembly. Since CAT strategy has a higher tolerance to high DNA concentration without formation of by-products as long as every junction is specific, it should have wide application in multiple-fragments ligation/assembly.

In summary, this study successfully established the CAT strategy to avoid the long-standing concentration dilemma in circular DNA construction. CAT was shown to significantly improve recombinant DNA, SLIC, USER and Gibson isothermal assembly, thus it can be applied to existing circular plasmid construction methods.

## Materials and methods

### Bacterial strains, media and plasmids

*E. coli* S17-1 λ*pir* [33-35] and *E. coli* S17-1 λ*pir*Δ*attB*_HK022_ were used as ligation and cyclization hosts (Table 3). The inactivation of HK022 *attB* [36] in *E. coli* S17-1 λ*pir* genome was carried out based on Wanner’s method of one-step inactivation of chromosomal genes [37]. The inactivation primers were listed in Additional file 4: Table S3. *E. coli* strain TSA was used as a linear plasmid host [22]. *Aeromonas hydrophila* 4AK4 [38] was used as the template for ligation of multiple fragments (F_1_…F_n_) (Table 3). Luria-Bertani (LB) broth was used to incubate various strains of *E. coli*. The *E. coli* strains harboring temperature-sensitive plasmid pAH83 [31] or pAH83CI [39,40]were incubated at 30°C. All other *E. coli* strains were incubated at 37°C. 25 mg/ml kanamycin, 50 mg/ml ampicillin or 25 mg/ml chloramphenicol were added to the cultures for maintaining or selecting *E. coli* harboring corresponding antibiotic resistance genes. All antibiotics were purchased from Sigma (Beijing, China).

**Table 3:** **Bacterial strains and plasmids used in this study**

**Strains and plasmids**	**Description**	**Reference(s) and/or derivations**
***E. coli*** **S17-1λ** ***pir***	TpR SmR recA, thi, pro, hsdR-M + RP4: 2-Tc:Mu: Km Tn7 λp	19
***E. coli*** **BL21(DE3)**	F^−^, ompT, hsdS(r_b_^−^m_b_^−^), gal, dcm(DE3)	Transgene Corporation
***E. coli*** **TSA**	Host strain fro linear plasmid	Lucigen corporation
***A. hydrophila*** **4AK4**	Amp^R^; Wild type isolated from sewage samples, PHBHHx producing strain	22
***P. denitrificans***	Gram-negative facultative anaerobic bacterium that performs denitrification, producing VB12	Huabei Pharmaceutical Enterprise
**pUK**	HK022 *attP* site, Km^R^	23
**pUKG**	pUK derived, *attL,attR* sequence produced by recombination, Km^R^	This study
**pCP20**	FLP recombinase helper plasmid, ts-rep, AmpR,Cm^R^	21
**pAH83CI**	pAH83 derived, expression control gene *cI* was broken by HindIII digesition	23
**pAH69**	pAL2 derived,*int* under the control of P1 promoter, expression control gene *cI* Amp^R^	*E.coli* Genetic Stock Center
**pJAZZOC**	Cloning vector	Lucigen corporation
**pJAZZG**	pJAZZOC derived, a fragment containing DraIII recognition site inserted.	This study
**pVQL**	P15A replicon, Cm^R^	Qing Lan corporation
**p15ABCDAI**	pVQL derived, haboring genes *hemBCD, cobAI*, Cm^R^	This study

In this study, HK022 *attL-attR* (Additional file 5: Table S4) recombination system was used for *in vivo* cyclization [30]. Plasmid pUKG (Additional file 6: Figure S2) was used as a template for vector backbone fragments F_R_, *attL-R6K* (*R6K*: R6kγ origin) and F_K_, *attR-Km* (*Km*: kanamycin resistant gene)*.* pUKG was constructed by the following procedures: pUK plasmid [39] was integrated into *E. coli* genome with the help of pAH69 [31] by recombination between *attB and attP*. The resulting *attL-R6K–Km-attR* fragment in the genome was then amplified by PCR, and self-ligated into a circular plasmid, namely pUKG (Table 3).

### Preparation of DNA fragments with sequence of 5 specific non-palindromic restriction endonucleases

In order to ligate N DNA fragments i.e. F_1_, F_2_ … F_N_, two fragments as backbone of the plasmids, namely, F_R_ and F_K_, were required. Totally N + 2 fragments were produced using PCR. The backbone fragments were different due to the various types of vectors and ligation methods used. In circular ligation, the F_R_ fragment contained *R6K* (R6kγ replicon) and *attL*, and the F_K_ fragment consisted of kanamycin resistant gene and *attR (attL* and *attR* were the HK022 *attL/R* recombination system*)*. Five non-panlindromic restriction sites (*Alwn*I, *Bgl*I, *Dra*III, *Pflm*I and *Sfi*I) for unidirectional ligation (i.e. each junction in the ligation is designed to be specific) were attached to all ends of the N + 2 fragments. For linear ligation, all the N + 2 fragments were the same except for no restriction site added at the *attL* and *attR* sides of F_R_ and F_K_. Furthermore, the N DNA fragments were ligated into the specific linear plasmid pJAZZ-OC-*Dra*III which was modified on the basis of pJAZZ-OC (Lucigen, USA) by inserting a fragment subcloned from pBBR1MCS2 [41] with two *Dra*III recognition sites. The two backbone fragments F_R_ and F_K_ were different, they are the two large digestion products of pJAZZ-OC-*Dra*III: F_R_ contained the linear replication origin, telN: telemorease and one hairpin end, and F_K_ was the chloramphenicol resistant gene linked with the other end of the hairpin [42]. All the fragments were digested by corresponding restriction enzymes, respectively, followed by purification using agarose gel-electrophoresis or solution extraction, and finally they were adjusted to a final molar concentration of 200 fmol/μl.

### *In vitro* ligation

Two ligation systems were adopted. The first one combined all the N + 2 fragments with the same molar concentration into one test tube, followed by the ligation process using the commercial Takara ligation kit. The second one is called “grouped ligation”, in which the linear ligation process was divided into several runs. Taking the group of ligation fragments F_R_, F_1_, F_2_, F_3_ and F_K_ as an example (Figure 5), F_R_ and F_1_ were ligated into F_R1_, F_2_ and F_3_into F_23_ in the first run, and F_K_ was left for the next run. During the second run of ligation, F_R1_ and F_23_ were ligated to form F_R123_, and F_K_ was left for the next round. In the final round, F_R123_ and F_K_ were ligated to become the final product F_R123K_. In each run, an equal mole of every two adjacent fragments was assigned in one group and ligated at 16°C for 2 h. This method could reduce unspecific ligation between non-adjacent fragments. After the *in vitro* ligation process, the circular DNA products that could replicate in *E. coli* directly were formed in circular ligation system, and *E. coli* S17-1λ*pir* was transformed with these circular DNA products.

### *In vitro* and *in vivo* cyclization

In the case of linear ligation system, the linear ligation products need to be cyclized to be able to replicate as circular plasmids in *E. coli*. In this study, three systems were adopted. The first was *in vitro* “diluted ligation cyclization”, the ligation products were diluted 10 folds with water in order to ensure self-ligation, and T4 ligase was used to cyclize the ligation products (The first branch from left of Figure 3C); The second one is “*in vitro* diluted self-recombination cyclization”, LR clonase was applied to cyclize the ligation products. In the case of LR clonase, the HK022 *attL* and *attR* in F_R_ and F_K_ were changed to λ*attL* and *attR*. All the *in vitro* cyclized products were electroporated into *E. coli* S17-1 λ*pir* electro-competent cells (The second branch from left of Figure 3C). Transformants were sprayed onto kanamycin agar plates, and then verified by PCR after 18 h of incubation. The last one is the “CAT strategy”, the linear ligation products were directly electroporated into electro-competent cells of *E. coli* S17-1 λ*pir*Δ*attB*_HK022_ carrying the corresponding pAH83CI helper plasmid (The right branch of Figure 3C). The pAH83CI could catalyze the recombination reaction of the Hk022 *attL/R* system.

### CAT on Recombinant DNA: Recombinant DNA using CAT

When CAT (Linear assembly - Transformation –*in vivo* Cyclization) is applied to a Recombinant DNA technology ligation of 3 fragments, the backbone fragment with an *attP* site is designed as 2 separate fragments with the *attL* and *attR* sites respectively for *in vivo* cyclization. The *attL* and *attR* sites will recombine and produce an *attP* site so that the final product is exactly the same as the expected ligation product from 3 fragment (Figure 4A). The fragments for digestion were all prepared by PCR. In the *in vitro* linear ligation of the CAT strategy, 4 DNA fragments digested by 5 specific non-palindromic restriction endonucleases were mixed at equal molar ratio to reach final total DNA concentrations of 0.5 pM, 1.0 pM, 2.0 pM, and then ligated into linear form intermediates by grouped ligation. And then the ligation products were directly electroporated into electro-competent cells of *E. coli* S17-1 λ*pir*Δ*attB*_HK022_ carrying the corresponding pAH83CI helper plasmid to do the *in vivo* cyclization step. At the meanwhile, 3 fragments digested by the same set of restriction enzymes were mixed at 0.5 pM, 1.0 pM, 2.0 pM concentrations and directly ligated into circular forms and then electroporated into the same *E. coli* S17-1 λ*pir*Δ*attB*_HK022_ competent cells carrying the corresponding pAH83CI helper plasmid to serve as the non-CAT control. The colonies on the Km screening plates from both CAT and non-CAT control sets were counted and verified by PCR and sequencing.

### CAT-Golden Gate: Golden Gate Assembly using CAT

Similarly, the CAT strategy was also applied to the Golden Gate methods. *Dra*III and *Bgl*I were used as the cutting enzymes in Golden Gate method. For short, in CAT strategy, 4 PCR fragments were mixed at equal molar ratio with 0.5 μl of *Dra*III (Fermentas), 0.5 μl of *Bgl*I (Fermentas), 5 units of T4 DNA ligase and 1 μl of T4 DNA ligase buffer and finally calibrated to a 10 μl digestion-ligation system with total DNA concentrations at 0.5 pM, 1.0 pM and 2.0 pM. The 3 fragments for non-CAT control were treated in the same way to reach the 0.5 pM, 1.0 pM and 2.0 pM digestion-ligation system, respectively. The digestion-ligations were performed in a thermocycler. Programs consist of the following steps: 1) incubation for 2 minutes at 37°C and 5 minutes at 16°C, both steps repeated 50 times; 2) incubation for 5 minutes at 50°C (final digestion); 3) 5 minutes at 80°C (heat inactivation). Finally, electro-competent cells of *E. coli* S17-1 λ*pir*Δ*attB*_HK022_ harboring the corresponding pAH83CI helper plasmid were transformed with the digestion-ligation products to finished the *in vivo* cyclization step. The colonies of transformants on the Km screening plates were counted and verified by PCR and sequencing.

### CAT-SLIC: SLIC using CAT

In a similar manner, CAT was also applied to SLIC. The 4 fragments for CAT-SLIC and 3 fragments for non-CAT control were prepared from the same templates as the recombinant DNA group, but a 40 bp homologous region that could specifically recombine under T4 DNA polymerase and annealing treatments were attached to each end. These PCR products were purified by gel-electrophoresis, and subsequently treated by T4 DNA polymerase for 5 minutes in a thermocycler. T4 DNA polymerase was inactivated at 75°C for 10 minutes, then, 4 fragments for CAT strategy were mixed together in a reaction system containing RecA and T4 ligation buffer at final DNA concentration of 0.5 pM, 1.0 pM and 2.0 pM, respectively. The annealing process was conducted by gradually reducing the temperature from 75°C to 37°C at the rate of 0.1°C/s. The 3 fragments for non-CAT control were treated in the same way. Finally, *E. coli* S17-1 λ*pir* was transformed with the reaction system and spread on a selective plate containing *Km.* The colonies of transformants were counted and verified by PCR and sequencing.

### CAT-Gibson: Gibson isothermal assembly using CAT strategy

Similar to the CAT-SLIC strategy, the 4 PCR fragments for CAT-Gibson or the 3 fragments for non-CAT Gibson control were mixed at final DNA concentrations of 0.5 pM, 1.0 pM and 2.0 pM in the Gibson Assembly Master Mix (2X). Then electro-competent cells of *E. coli* S17-1 λ*pir*Δ*attB*_HK022_ harboring the corresponding pAH83CI helper plasmid were transformed with the Gibson assembly products to complete the *in vivo* cyclization step. The colonies of transformants on the Km screening plates were counted and verified by PCR and sequencing.

### Construction of optimized precorrin-3A synthesis pathway from precursor 5-aminolevulinic acid

The genes for precorrin-3A synthesis pathway from precursor 5-aminolevulinic acid include *hemB, hemC, hemD, cobA,* and *cobI.* Among these genes, *hemB, hemC, hemD* are from *Bacillus subtilis. cobA, cobI* from *P. denitrificans* (Table 3). For the expression of these 5 genes in *E. coli*, codon optimization was conducted. These genes were synthesized chemically. These 5 genes were constructed into one plasmid p15ABCDAI which requires a multiple DNA (in this case, 7) fragments assembly method. These 7 fragments include F_R_ (*attL-*p15a-T7), F_1_ (*hemB*), F_2_ (*hemC*), F_3_ (*hemD*), F_4_ (T7-*cobA*), F_5_ (*cobI*) and F_k_ (*Cm-attR*).

The F_R_ fragment was prepared by over-extension PCR using the template plasmid pUK and pVQL, the T7 promoter was attached into the primer sequence_._ The 7 fragments were cloned, digested, and linearly ligated in group according the standard grouped *in vitro* linear ligation method (Figure 5B). Then, electro-competent cells of *E. coli* S17-1 λ*pir*Δ*attB*_HK022_ harboring the corresponding pAH83CI helper plasmid were transformed with the ligation products (The right branch of Figure 3C). Transformants were sprayed onto chloromycetin agar plates, and then verified by PCR after 18 h of growth. The *E. coli* BL21 (DE3) was transformed with the constructed plasmid p15ABCDAI to from the strain *E. coli* BL21DE3 (p15ABCDAI). The BL21DE3 (p15ABCDAI) strain was cultured by LB medium with 1 g/L 5-aminolevulinic acid as precursor to test the production of precorrin 3A.

## Additional files

Additional file 1: Table S1. Primers for 4 and 6 fragments used for *in vitro* circular ligation and linear ligation. Additional file 2: Table S2. Primers for 9 fragments linear ligation. Additional file 3: Figure S1. Demonstration of cyclization. Additional file 4: Table S3. Primers used for gene knock out and verification for HK022 *attB* site. Additional file 5: Table S4. Sequences of HK022 *attL* and HK022 *attR*. Additional file 6: Figure S2. Plasmid map of pUKG.
