# Supplementary material for: A strategy for enhanced circular DNA construction efficiency based on DNA cyclization after microbial transformation
Source: Microb Cell Fact. 2015 Feb 12;14:18. doi: 10.1186/s12934-015-0204-x (PMC4455692; doi:10.1186/s12934-015-0204-x)
Supplement: Additional file 1: Table S1. — Primers for 4 and 6 fragments used for in vitro circular ligation and linear ligation. [file 12934_2015_204_MOESM1_ESM.doc]

**Additional file 1: Table S1: Primers for 4 and 6 fragments used for *in vitro*** circular ligation and linear ligation

| **Primer for fragments** | **Sequences** |
| --- | --- |
| F_R_F | TACTGGTCAGGGTTAACAG |
| F_R_R | ATCCAGATcacctagtgGCCTTCTTGACGAGTTCTT |
| F_1_F | AATTGCATccagtagttggGTCCGAGGATACAAAGTGCGTTCA |
| F_1_R | AATTGCATccaatcactggCCTTGCCGCCATACCAGAGC |
| F_2_F | AATTGCATccagtgattggCCGAGGATACAAAGTGCGTTCAT |
| F_2_R | AATTGCATccaaagcctggCCCTTGCCGCCATACCAGA |
| F_3_F | AATTGCATgccggcttggcGACGCCTCTTACTTTCTGGTCTACG |
| F_3_R | AATTGCATgccaacccggcACCCATCCACAACTCAAGCCAC |
| F_4_F | AATTGCATgccgggttggcCGCCTCTTACTTTCTGGTCTACGG |
| F_4_R  F_K_4R: | AATTGCATgccaagtcggcACCCATCCACAACTCAAGCCAC  CGATAAAT ccaagctctgg CGTACTATCAACAGGTTGAAC |
| F_K_F | ACGCGCATGATAGCCTCA |
| F_K_6R | ATCGATAA ccaaactctgg CGTACTATCAACAGGTTGAAC |
| F_RC_F | AATTGCATccaacgactgg TACTGGTCAGGGTTAACAG |
| F_KC_R | AATTGCATccagtcgttgg ACGCGCATGATAGCCTCA |

* The underlines indicate recognition sites for 5 types of different endonucleases. The red color indicates sticky ends.
