# Supplementary material for: A strategy for enhanced circular DNA construction efficiency based on DNA cyclization after microbial transformation
Source: Microb Cell Fact. 2015 Feb 12;14:18. doi: 10.1186/s12934-015-0204-x (PMC4455692; doi:10.1186/s12934-015-0204-x)
Supplement: Additional file 2: Table S2. — Primers for 9 fragments linear ligation. [file 12934_2015_204_MOESM2_ESM.doc]

**Additional file 2: Table S2: P**rimers for 9 fragments linear ligation

| **Primers for fragments** | **Sequences** |
| --- | --- |
| F_R_F | TACTGGTCAGGGTTAACAG |
| F_R_R | AATAAATA gccaggtaggc GCCTTCTTGACGAGTTCTT |
| F_1_F | ATCATTAAcacaccgtgGCATCTGGAAGGGACGCT |
| F_1_R | TCAACTGTcaccgggtgGCTGGCTCTGGTGGGC |
| F_2_F | ATCTGGTAgccaccgaggcTTGGAGGCTGGAAATCTATGC |
| F_2_R | GTCTATCTgccatgaaggcGGTGCTGTCGTTGGTGTTG |
| F_3_F | ATGTGCTTcactcagtgCGTAGGCGGTGGCGG |
| F_3_R | TAATAGTTcacgaagtgCGGGAGATGGACAGCGA |
| F_4_F | ACATAATCgccattcaggcGCTCATCTCCTCGTTGCCA |
| F_4_R | ACCTCCAGgccatacaggcGACCGTTTTCGCTTTACCG |
| F_5_F | TCACCCAGcacgtagtgTTAGGCAACGGCAAATGAAT |
| F_5_R | CTCCACAAcacatggtgCTGATGATGACGGTGAAGCG |
| F_6_F | ATAATTATgccacataggcCTGGATGTGGTGGATGCCC |
| F_6_R | ACATAAAAgccagctaggcGGAGACCGAATACTGCTGCG |
| F_7_F | ATAAAAATcacagcgtgCCCTGCCCTTCCTGCTG |
| F_7_R | ACAAATTTcacaaggtgCGCCTGACCCATCCACA |
| F_K_R | TTATATAgcctctttggcCCGTACTATCAACAGGTTGAAC |
| F_K_F | ACGCGCATGATAGCCTCA |

* The underlines indicate recognition sites for 5 types of endonucleases. The red color indicates sticky ends.
