# Supplementary material for: A strategy for enhanced circular DNA construction efficiency based on DNA cyclization after microbial transformation
Source: Microb Cell Fact. 2015 Feb 12;14:18. doi: 10.1186/s12934-015-0204-x (PMC4455692; doi:10.1186/s12934-015-0204-x)
Supplement: Additional file 3: Figure S1. — Demonstration of cyclization. [file 12934_2015_204_MOESM3_ESM.doc]

**Additional file 3: Figure S1: Demonstration of cyclization**

**
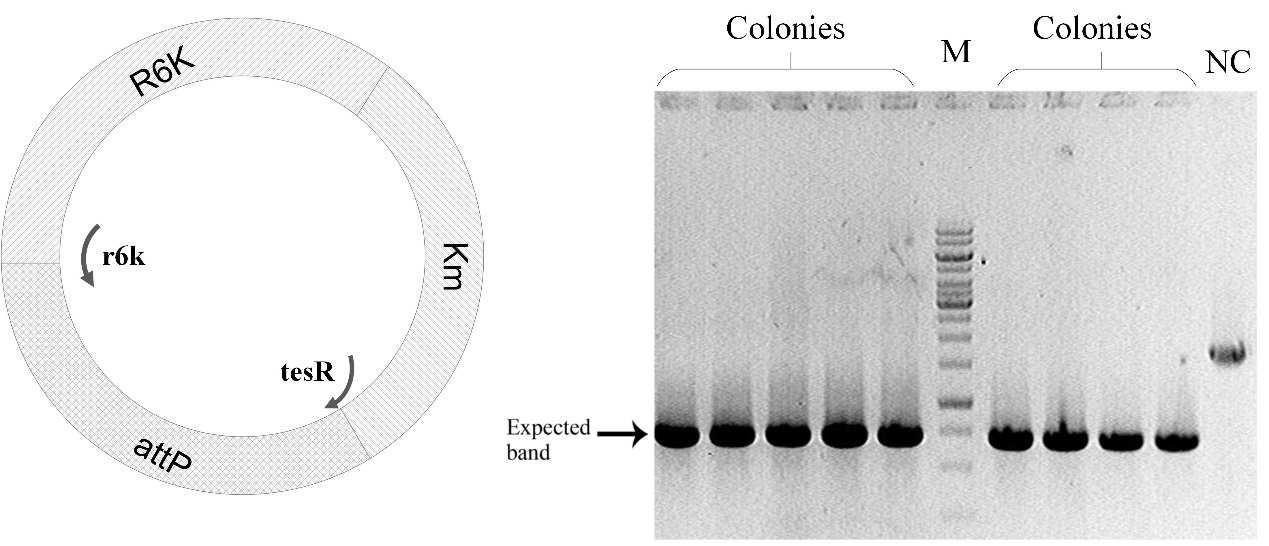
**

The band in the last lane of electrophoresis significantly above the other band was a negative control which appeared when recombination did not occur.
