# Supplementary material for: A strategy for enhanced circular DNA construction efficiency based on DNA cyclization after microbial transformation
Source: Microb Cell Fact. 2015 Feb 12;14:18. doi: 10.1186/s12934-015-0204-x (PMC4455692; doi:10.1186/s12934-015-0204-x)
Supplement: Additional file 4: Table S3. — Primers used for gene knock out and verification for HK022 attB site. [file 12934_2015_204_MOESM4_ESM.doc]

**Additional file 4: Table S3: Primers used for gene knock out and verification for HK022 *attB* site**

| **Primers for knock out and**  **verification** | **Sequence** |
| --- | --- |
| attBF | TGGTAGGAATCAATGCCTGAGTGTTGTCTTTTTCCACCTATTCCGGGGATCCGTCGACC |
| attBR | GCGGCGTAGAGGCTTGCACCGTGAAATGCTGCGCATCATTGTAGGCTGGAGCTGCTTCG |
| attBVF | GAATCAATGCCTGAGTGTTGTCTT |
| attBVR | TCGGGAAATGAGGTCGTGCTA |
