# Supplementary material for: A strategy for enhanced circular DNA construction efficiency based on DNA cyclization after microbial transformation
Source: Microb Cell Fact. 2015 Feb 12;14:18. doi: 10.1186/s12934-015-0204-x (PMC4455692; doi:10.1186/s12934-015-0204-x)
Supplement: Additional file 5: Table S4. — Sequences of HK022 attL and HK022 attR. [file 12934_2015_204_MOESM5_ESM.doc]

**Additional file 5: Table S4: Sequences of HK022 *attL* and HK022 *attR***

| **Sequence name** | **Sequence** | |
| --- | --- | --- |
| HK022 *attL* | | TTAAATTCACGGTCGGTGCACTTTAGGTGAATAAGTTGTATATTTAAAATCTCTTTAATTATCAGTAAATTAATGTAAGTAGGTCATTATTAGTCAAAATAAAATCATTTGTCGATTTCAATTTTGTC |
| HK022 *attR* | | TCAGGTCACTAATACTATCTAAGTAGTTGATTCATAGTGACTGGATATGTTGCGTTTTGTCGCATTATGTAGTCTATCATTTAACCACAGATTAGTGTAATGCGATGATTTTTAAGTGATTAATGTTATTTTGTCATCCTTTAGGTGAAAAAGGTTGAGTCGCAAAGCG |
