# Supplementary material for: A strategy for enhanced circular DNA construction efficiency based on DNA cyclization after microbial transformation
Source: Microb Cell Fact. 2015 Feb 12;14:18. doi: 10.1186/s12934-015-0204-x (PMC4455692; doi:10.1186/s12934-015-0204-x)
Supplement: Additional file 6: Figure S2. — Plasmid map of pUKG. [file 12934_2015_204_MOESM6_ESM.doc]

**Additional file 6: Figure S2: Plasmid map of pUKG**

Raw sequence for pUKG

atgagatcgcacgcctggcgcaaggtcaggcgaataatgcgacaacttgaaccagggcacaccaacgcggcttttcacctggggagcatcaatagcatttaccagttcgatcaccggcagacttgctacctgcttttgcagatcgggaaatgaggtcgtgctactaccgagtaaaatggcctctgcgccccactgtttacactggtcgatttgtgcttgctgggtagccaactggctgtagccgcctgcctccagcacttttaaatccacaccatagcggcgagctgcctcctgcataccatagttcaacgataaccagtatgaatctttcaggctgggataaagcgcgcacagtttccatgcgcgtttggctttaagcggcatagaggcttgcaccgtgaaatgctgcgcatcatgccagcgcaacaggttatcagccgaaaatgccggcaacatgaaaagggaaagaagtaaaaatagcagtacgcgcatgatagcctcatcaataataaggctttatgctagatgcattccgctttgcgactcaacctttttcacctaaaggatgacaaaataacattaatcacttaaaaatcatcgcattacactaatctgtggttaaatgatagactacataatgcgacaaaacgcaacatatccagtcactatgaatcaactacttagatagtattagtgacctgagacagagcattagcgagctcagatgaattcaatctctagaagagcgcttttgaagctcacgctgccgcaagcactcagggcgcaagggctgctaaaggaagcggaacacgtagaaagccagtccgcagaaacggtgctgaccccggatgaatgtcagctactgggctatctggacaagggaaaacgcaagcgcaaagagaaagcaggtagcttgcagtgggcttacatggcgatagctagactgggcggttttatggacagcaagcgaaccggaattgccagctggggcgccctctggtaaggttgggaagccctgcaaagtaaactggatggctttcttgccgccaaggatctgatggcgcaggggatcaagatctgatcaagagacaggatgaggatcgtttcgcatgattgaacaagatggattgcacgcaggttctccggccgcttgggtggagaggctattcggctatgactgggcacaacagacaatcggctgctctgatgccgccgtgttccggctgtcagcgcaggggcgcccggttctttttgtcaagaccgacctgtccggtgccctgaatgaactgcaggacgaggcagcgcggctatcgtggctggccacgacgggcgttccttgcgcagctgtgctcgacgttgtcactgaagcgggaagggactggctgctattgggcgaagtgccggggcaggatctcctgtcatctcaccttgctcctgccgagaaagtatccatcatggctgatgcaatgcggcggctgcatacgcttgatccggctacctgcccattcgaccaccaagcgaaacatcgcatcgagcgagcacgtactcggatggaagccggtcttgtcgatcaggatgatctggacgaagagcatcaggggctcgcgccagccgaactgttcgccaggctcaaggcgcgcatgcccgacggcgaggatctcgtcgtgacccatggcgatgcctgcttgccgaatatcatggtggaaaatggccgcttttctggattcatcgactgtggccggctgggtgtggcggaccgctatcaggacatagcgttggctacccgtgatattgctgaagagcttggcggcgaatgggctgaccgcttcctcgtgctttacggtatcgccgctcccgattcgcagcgcatcgccttctatcgccttcttgacgagttcttctaatctcgagaaaacttcatttttaatttttgcggccgcaagatccgcagttcaacctgttgatagtacgtactaagctctcatgtttcacgtactaagctctcatgtttaacgtactaagctctcatgtttaacgaactaaaccctcatggctaacgtactaagctctcatggctaacgtactaagctctcatgtttcacgtactaagctctcatgtttgaacaataaaattaatataaatcagcaacttaaatagcctctaaggttttaagttttataagaaaaaaaagaatatataaggcttttaaagcttttaaggtttaacggttgtggacaacaagccagggatgtaacgcactgagaagcccttagagcctctcaaagcaattttcagtgacacaggaacacttaacggctgacatgggaattagctatggcaaagcctcgcaatccccatcctttttcgcacgatatacaggattttgccaaagggttcgtgtagactttccttggtgtatccaacggcgtcagccgggcaggataggtgaagtaggcccacccgcgagcgggtgttccttcttcactgtcccttattcgcacctggcggtgctcaacgggaatcctgctctgcgaggatccatgggacaaaattgaaatcgacaaatgattttattttgactaataatgacctacttacattaatttactgataattaaagagattttaaatatacaacttattcaccttaagtgcaccgaccgtgaatttaaccctgacccgaagactctggatgggctttgccctgatggcgctgttaaccctgaccagtaccctggtgggatggtacaacctgcgctttatcagccaggtggaaaaagacaacactcaggcattgattcctaccatgaatatggcgcgccagttgagcgaagccagcgcctgggaacttttcgccgcgcagaacctgaccagtgccgataacgaaaagatgtggcaggcgcaggggcgaatgctcaccgcacaaagcctgaagattaatgcgttgctgcaagcgttacgggaacaaggttttgataccaccgctattgaacaacaggagcaggagatctcccgttcattacgtcagcaaggggaactggtggggcggcgtctgcaactacgccagcaacaacggcaactcagtcagcagatagtcgctgccgccg
